# The association between second to fourth digit ratio, reproductive and general health among women: findings from an Israeli pregnancy cohort

**DOI:** 10.1038/s41598-020-62599-3

**Published:** 2020-04-14

**Authors:** Maya Tabachnik, Eyal Sheiner, Tamar Wainstock

**Affiliations:** 1 The Department of Public Health, Faculty of Health Sciences, Ben-Gurion University of the Negev, POB 653, Beer-Sheva, 84105 Israel; 2 Department of Obstetrics and Gynecology, Soroka University Medical Center, Ben-Gurion University of the Negev, POB 151, Beer-Sheva, 84101 Israel

**Keywords:** Intrauterine growth, Environmental impact, Epidemiology

## Abstract

The ratio between the length of second and fourth digits (2D:4D) is a putative biomarker for prenatal testosterone and estrogen exposure. The aim of the study was to examine the association between 2D:4D and women’s general and reproductive health. This analysis was conducted within a prospective pregnancy cohort study. The study population included 187 women. 2D:4D was measured directly in both hands using a digital caliper. Multivariable linear and logistic models were used to study the associations between digit ratio and the studied health characteristics. Mean age of the participants was 30.7 ± 4.9 years. The mean age at menarche was 12.9 ± 1.4 years. Right hand 2D:4D mean ± SD was 0.965 ± 0.03. Left hand 2D:4D mean ± SD was 0.956 ± 0.03. An association was found between right 2D:4D and age at menarche, with older age in women with 2D:4D ≥ mean versus 2D:4D < mean (13.2 ± 1.5 and 12.8 ± 1.3 respectively, b = 0.48, 95%CI:0.06–0.91) while controlling for ethnicity. Higher 2D:4D was also associated with heavier menses bleeding and dysmenorrhea. There is an association between 2D:4D and sub optimal reproductive characteristics, including later age at menarche, heavier menses bleeding and dysmenorrhea. These findings support the association between the intrauterine period and reproductive characteristics. Further studies are required to support our findings.

## Introduction

The ratio between lengths of the second and the fourth digits (index and ring fingers) (2D:4D) is a putative biomarker of prenatal testosterone and estrogen exposure^1^. 2D:4D is sexually dimorphic, and males’ 2D:4D is relatively shorter than females’^2,3^. 2D:4D is observed at as early as the end of the first trimester^4,5^. Relative digit length is considered steady at ages 2 to 25^6^, although other studies suggest that 2D:4D does mildly increase during childhood but remains steady during puberty^7,8^.

2D:4D was suggested to be associated with Hox gene family^2,3^. Homeobox genes Hox a and Hox d may indirectly influence the prenatal production of androgen by controlling the differentiation of the urogenital system as well as digit development. Considering this, it was suggested that there may be an association between prenatal hormonal function and digit formation, and 2D:4D was proposed as an indicator of first trimester testosterone and estrogen levels, reflecting the Hox genes’ action on differentiation^9,10^.

Environmental exposure to toxicants is critical during early development stages, such as the perinatal period, in which there is a heightened vulnerability to exposures due to potential permanent effect on health and development^11^. Previous research showed association between prenatal hormonal environment and exposure to endocrine disrupting chemicals and 2D:4D in animals^12,13^ and humans^2,10,14–16^. Conversely, some studies found no support for such association^17–19^.

Assessing the prenatal hormonal environment directly from the fetus in utero is challenging because of the risk for the fetus. Therefor other methods have been used to evaluate prenatal androgen exposure, such as 2D:4D^7^. Various studies found associations between 2D:4D and reproductive characteristics in humans, including age at menarche^20–23^, puberty characteristics^24^, reproductive period^20^, age at menopause^25^, polycystic ovary syndrome^26^, severity of premenstrual symptoms^27^ and estradiol levels across menstrual cycle^28,29^. Several studies found 2D:4D to be associated with reproductive cancers^25,30,31^. As for general health characteristics, 2D:4D was associated with various cancers^32,33^, idiopathic pulmonary hypertension^34^ and other morbidities such as systemic lupus erythematosus^35^, autism spectrum disorders^36^, and congenital adrenal hyperplasia^37,38^.

Not all studies however found an association between 2D:4D in women and reproductive^39–43^ and health characteristics^44,45^ and there is some inconsistency in the literature regarding the direction of the association. For instance, several large studies found a negative association between 2D:4D and age at menarche^20,22,23^, while another found a positive association^21^. Due to this inconsistency the goal of the current study was to assess the association between women’s general and reproductive health and 2D:4D. Moreover, in the present cohort we studied a new population in which 2D:4D was not yet researched – women from Israel. This study aims to clarify previously disparate findings regarding the association and direction of 2D:4D and reproductive and health characteristics in women. Furthermore, by inspecting a new population it also aims to replicate previously reported associations between 2D:4D and reproductive and general health characteristics.

## Methods

### Study design

This analysis was conducted in the framework of The Negev Pregnancy Cohort study.

### Setting

Study population included pregnant women from the Negev region in Israel. Women were recruited at a nuchal translucency clinic at Soroka University Medical Center (SUMC) to which pregnant women attend at 11–13.5 gestational weeks for a nuchal translucency ultrasound, which is a screening test for major chromosomal and nuchal cord abnormalities. These tests are routinely recommended for all pregnant women and are covered by national health insurance law, by the health maintenance organizations. Women with multi fetal pregnancies in the current pregnancy were excluded from the study. As the current study is a part of a prospective cohort study which is aimed to conduct a long term follow up on participants and their offspring, women who did not plan to give birth at SUMC were also excluded.

The study protocol was approved by SUMC IRB committee. All methods were performed in accordance with the relevant guidelines and regulations. Recruitment period occurred from October 2017 to April 2018. All eligible women were offered to participate in the study and signed an informed consent. Participants underwent anthropometric measurements and completed a comprehensive questionnaire with a trained interviewer, which included background and socioeconomic characteristics, environmental exposures and reproductive and general health characteristics. Women were compensated for their participation.

### Study variables

2D:4D was the independent variable, and was directly measured in both right and left hand using a digital caliper (resolution: 0.01 mm, manufactured by FST, Germany). Finger length was measured from the proximal palm crease to the fingertip as in Auger *et al*. who validated the method against hand radiographies^46^. Additional anthropometric measurements included weight, body mass index (BMI), waist and hip circumference. Height was self- reported. Each measurement was conducted twice by the same research assistant. If there was a difference of more than 10% between measurements, a third measure was taken and the two measurements with less than 10% difference were chosen for the analysis. The intraclass correlation coefficients between each measurement were high (rt. hand 2^nd^: r = 0.95; rt. hand 4^th^: r = 0.97; lt. hand 2^nd^: r = 0.96; lt. hand 4^th^r = 0.97; p < 0.001). The Kappa between each pair of measurements was also significant (p < 0.001). A mean of both measurements was calculated and used in the analysis. One observation of 2D:4D was excluded after a cutoff of an outlier (>2 standard deviations from the mean).

Data of background and outcome variables were obtained by comprehensive questionnaires. Background information included sociodemographic variables. Outcome variables included general and reproductive health characteristics. Reproductive information included age at menarche, cycle length, menses duration, menses bleeding degree (lighter than normal, normal, heavier than normal), dysmenorrhea (defined as menstrual pain and cramps), bleeding during current pregnancy, time to pregnancy, fertility treatments for current or previous pregnancy, complications in current pregnancy such as high blood pressure, diabetes and thyroid disorders etc. General health information included ever being told by a physician or diagnosed with any health problems, based on a pre-defined list of common morbidities Such as cardiovascular disease (CVD), Anemia, thyroid disorders, herpes, migraine/headache, faint, dizziness etc.

### Statistical analysis

The completed questionnaires were reviewed to verify competence. Data were de-identified, coded and entered using ACCESS data management software, and were analyzed using SPSS software (version 23.0).

In order to validate the completeness and veracity of some reported reproductive health characteristics, several questions were repeated and their answers compared in the questionnaires, including age at menarche, cycle length and time to pregnancy. Additionally, one woman with an extreme value of age at menarche of 7 was excluded from the age at menarche analysis. This woman had the following digit ratios: rt.: 0.974, lt.: 0.942.

Univariate association between 2D:4D (as a continuous variable and as above versus below mean) and outcome variables was considered with t-test for continuous outcome variables and Chi-square test for categorical outcome variables. A multivariate analysis was conducted between 2D:4D and the variables found in the univariable level to be associated with 2D:4D. All analyses were two-sided with an alpha of 5%.

### Ethics approval and consent to participate

The study protocol was approved by SUMC IRB committee (0272-16-SOR). All participants signed an informed consent form.

## Results

### Background information

A total of 187 women were included in the study. Participant’s age ranged between 20–42 years with mean age of 30.7 ± 4.9 years. The majority of the women, 83.1%, were Jewish and 13.4% were Muslims. All anthropometric measurements were characterized with a normal distribution. Right hand 2D:4D ranged between 0.852–1.064 with mean ± SD of 0.965 ± 0.03. Left hand 2D:4D ranged between 0.879–1.067 with mean ± SD of 0.956 ± 0.03. Table 1 presents selected background characteristics of the study population separately for each hand by above versus below mean digit ratio, including demographic and anthropometric information. As can be seen, the study groups did not differ in the background characteristics.

**Table 1:** Background characteristics of study population by above versus below mean digit ratio for each hand^a^.

Characteristic	Right 2D:4D Digit Ratio				Left 2D:4D Digit Ratio			
	Total N = 186^b^	<mean	≥ mean	OR 95% CI/p	Total N = 187	<mean	≥ mean	OR 95% CI/p
**Demographic**^c^								
**Age** mean (±SD)	171	30.2 (5.1)	31.3 (4.7)	0.14	172	30.5 (5.1)	31.0 (4.8)	0.46
**Ethnicity** Jewish	142 (83.0)	74 (78.7)	68 (88.3)	2.04 (0.87–4.79)	143 (83.1)	75 (79.8)	68 (87.2)	1.72 (0.75–3.96)
**Country of birth** Israel	146 (85.9)	82 (88.2)	64 (83.1)	0.66 (0.28–1.57)	147 (86.0)	84 (90.3)	63 (80.8)	0.45 (0.19–1.09)
**Marital status** Married	164 (95.9)	91 (96.8)	73 (94.8)	0.60 (0.13–2.77)	165 (95.9)	90 (95.7)	75 (96.2)	1.11 (0.24–5.12)
**Employment status** Employed	144 (85.2)	79 (84.9)	65 (85.5)	1.05 (0.45–2.46)	145 (85.3)	75 (80.6)	70 (90.9)	2.40 (0.95–6.09)
**Anthropometric**								
Height mean (±SD)	186	162.4 (5.5)	161.9 (6.4)	0.61	187	162.2 (5.9)	162.1 (5.8)	0.97
BMI mean (±SD)	186	25.6 (5.3)	25.6 (4.9)	0.91	187	26.0 (5.3)	25.2 (4.7)	0.26
Waist mean (±SD)	186	84.0 (10.2)	83.9 (10.7)	0.94	187	84.9 (10.5)	82.8 (10.2)	0.17
Thighs mean (±SD)	186	104.7 (10.8)	104.3 (10.8)	0.78	187	105.2 (10.7)	103.7 (10.8)	0.34
Waist:Thighs mean (±SD)	186	0.8 (0.1)	0.8 (0.1)	0.74	187	0.8 (0.1)	0.8 (0.1)	0.38

^a^All numbers presented as n (%), unless otherwise stated.

^b^one outlier was excluded.

^c^demographic variables with missing data for 16 women.

### Reproductive and general health characteristics

Table 2 presents reproductive and general health characteristics separately for each hand by above versus below mean digit ratio. The mean cycle length was 30.1 ± 12.3 days, the mean menses duration was 5.1 ± 1.5 days and the mean age at menarche was 12.9 ± 1.4 years. Age at menarche was associated with right 2D:4D and the correlation between age at menarche and 2D:4D (continuous format) was r (Pearson) = 0.17 (p = 0.034).

**Table 2:** Reproductive and general health characteristics of study population by above versus below mean digit ratio^a^.

Characteristic	Right				Left			
	2D:4D Digit Ratio				2D:4D Digit Ratio			
	Total N = 170^b^	<mean	≥mean	OR 95% CI/p	Total N = 171	<mean	≥mean	OR 95% CI/p
**Infertility**^**c**^	33 (20.0)	19 (20.9)	14 (18.9)	0.88 (0.41–1.91)	33 (19.9)	19 (20.7)	14 (18.9)	0.90 (0.42–1.94)
**Bleeding during pregnancy**	29 (17.1)	11 (11.8)	18 (23.4)	2.27 (1.00–5.17)	29 (17.0)	11 (11.8)	18 (23.1)	2.24 (0.98–5.08)
**Menses characteristics**								
**Age at menarche (years)** mean (±SD)	164	12.8 (1.3)	13.2 (1.5)	**0.04**	165	12.8 (1.4)	13.1 (1.4)	0.21
**Cycle length (days)**^**d**^ mean (±SD)	115	29.6 (5.3)	30.8 (17.9)	0.59	116	30.9 (15.9)	29.2 (5.6)	0.47
**Menses duration (days)**^**d**^ mean (±SD)	103	5.1 (1.7)	5.1 (1.4)	0.95	103	5.3 (1.7)	4.9 (1.4)	0.29
**Menses bleeding**^**d**^ Heavier than normal	12 (7.1)	5 (5.5)	7 (9.1)	1.72 (0.52–5.66)	12 (7.1)	8 (8.8)	4 (5.1)	0.56 (0.16–1.94)
**Dysmenorrhea**^**d**^	66 (63.5)	37 (60.7)	29 (67.4)	1.34 (0.59–3.05)	66 (63.5)	33 (55.9)	33 (73.3)	2.17 (0.94–5.00)
**General health characteristics**^**e**^								
**Anemia**	50 (29.6)	27 (29.3)	23 (29.9)	1.03 (0.53–1.99)	50 (29.4)	31 (33.7)	19 (24.4)	0.63 (0.32–1.24)
**CVD**^**f**^	16 (9.4)	7 (7.5)	9 (11.7)	1.63 (0.58–4.59)	16 (9.4)	6 (6.5)	10 (12.8)	2.13 (0.74–6.16)
**Thyroid disorders**^**g**^	21 (12.4)	12 (12.9)	9 (11.7)	0.89 (0.36–2.25)	21 (12.3)	11 (11.8)	10 (12.8)	1.10 (0.44–2.74)
**Herpes**	22 (12.9)	12 (12.9)	10 (13.0)	1.01 (0.41–2.48)	22 (12.9)	11 (11.8)	11 (14.1)	1.22 (0.50–3.00)
**Migraine/Headache**	63 (37.3)	35 (38.0)	28 (36.4)	0.93 (0.50–1.74)	63 (37.1)	35 (38.0)	28 (35.9)	0.91 (0.49–1.71)
**Faint**	17 (10.0)	10 (10.8)	7 (9.1)	0.83 (0.30–2.30)	17 (9.9)	9 (9.7)	8 (10.3)	1.07 (0.39–2.91)
**Dizziness**	33 (19.4)	20 (21.5)	13 (16.9)	0.74 (0.34–1.61)	33 (19.3)	21 (22.6)	12 (15.4)	0.62 (0.29–1.37)

^a^All numbers presented as n (%), unless otherwise stated.

^b^one outlier was excluded.

^c^Infertility defined as having at least one of the following: endometriosis, time to pregnancy >1 year, any fertility treatment in current or previous pregnancy.

^d^Including only women who did not use contraceptives in the last year.

^e^Data presented include variables with at least 10% women with the outcome.

^f^Cardiovascular disease (CVD) defined as having at least one of the following: hypertension, cholesterol or triglycerides, congestive heart failure or stroke, hypertension in current pregnancy.

^g^Thyroid disorders defined as having at least one of the following: hypothyroidism or hyperthyroidism throughout life, hypothyroidism, hyperthyroidism or other thyroid disorder in current pregnancy.

Table 3 presents the multivariable analysis for the association between 2D:4D and age at menarche. As can be seen, there was a significant association between right 2D:4D and age at menarche, while controlling for ethnicity (b = 0.48, 95%CI 0.06–0.91).

**Table 3:** Multivariable analysis for the association between right 2D:4D, menses bleeding, dysmenorrhea and age at menarche.

	Heavy menses bleeding		Age at menarche^a^		Dysmenorrhea	
	Adjusted OR (95% CI)	p	b (95% CI)	p	Adjusted OR (95% CI)	p
2D:4D^b^ (above versus below mean)	2.49 (0.69–8.91)	0.16	0.48 (0.06–0.91)	0.03	2.34 (0.98–5.62)	0.05
Age (years)	0.91 (0.79–1.04)	0.15			0.89 (0.82–0.97)	0.01
Ethnicity (Jewish)	0.29 (0.08–1.11)	0.07	−0.41 (−0.97–0.15)	0.15	1.64 (0.56–4.81)	0.37

^a^Adjusted R^2^ = 0.03.

^b^Rt. hand 2D:4D for heavy bleeding and age at menarche, Lt. hand 2D:4D for dysmenorrhea.

Among women who complained about heavy bleeding during menstrual period, accompanied with cramps and abdominal pain (n = 66 out of the 104 which did not take birth control pills), digit ratio was significantly higher as compared to women with no such complaints (0.96 ± 0.03 versus 0.94 ± 0.02, p < 0.01). As can be seen in Table 3, although the direction of the association between 2D:4D and heavy bleeding remained, it was no longer significant in the multivariable analysis. Women who reported having dysmenorrhea were more likely to have higher left hand 2D:4D. This association was marginally significant in the multivariable analysis (also presented in Table 3). The models were tested separately among the larger ethnic group in the study population (Jewish women), and results remained similar.

As for general health characteristics, prevalence of all morbidities was not associated with 2D:4D, besides thyroid disorders, which were more common among women with higher 2D:4D.

## Discussion

In the current study an association was found between higher 2D:4D and sub-optimal reproductive characteristics, including: older age at menarche, heavy menses bleeding and dysmenorrhea.

Our study found a positive correlation between right 2D:4D and age at menarche, a result that replicates that of Oberg *et al*. who used a direct measurement of 2D:4D in a large study of 299 girls and found a lower median age at menarche in girls from the lowest 2D:4D tertile in comparing to the highest^21^. Older age at menarche may represent sub-optimal reproductive profile, and has been associated with subfecundity and infertility^47^. Additional studies reported an association between right 2D:4D and age at menarche, however the association they found was negative. Their findings are unlikely to have been underpowered. Kalichman *et al*. found in a sample of 674 women that women with 2D < 4D in both hands, had a higher age at menarche. The measurements were based on X-rays digitalized images^20^. Similarly, Manning *et al*. in a large population study of 70, 658 women found a negative association between right 2D:4D and age at menarche. Digit length measurements in that study were self-measured by the participants using a ruler^23^. Correspondingly, Matchock, in a study of 206 women, found that women with low right 2D:4D reported delayed age at menarche. Digit length measurements were obtained from photocopies^22^.

However, Muller *et al*. in a large study of 9,044 women^25^, Gooding *et al*. in a sample of 202 women^40^ and Helle in a study of 282 women^41^ found no association between 2D:4D and age at menarche. Digit length measurements in those studies were obtained from scans and photocopies. Likewise, Li *et al*.^24^ in a study of 318 girls found no association between 2D:4D and age at menarche using a direct measurement of the digit length. Nonetheless the study used a different measurement methodology than in the current study and examined only left hand digit ratio while left hand 2D:4D is less sensitive to prenatal hormonal environment^10,23^. Further studies with similar methodology are recommended to support the association and clarify its direction.

Lower 2D:4D in our population, which is characterized as a more masculine digit ratio, was associated with earlier age at menarche. On one hand younger age at menarche can mean that reproduction can begin earlier. It is noteworthy to acknowledge the Belsky *et al*. evolutionary theory of socialization that considers the timing of puberty as an outcome of social experience. Drawing from evolutionary biology contending that humans adapt to circumstantial conditions in ways that will increase reproductive competence, they suggest early menarche can be related to early stress through biological reaction to social conditions. They suggest that early puberty may shorten the period between puberty and actual fertility consequently organizing menstrual cycle in a manner that enables lifespan with more conceptions^48^.

Younger age at menarche can also mean the depletion of the eggs reserve earlier. In previous research, earlier age at menarche was associated with an increased risk for various morbidities later in life such as CVD, type 2 diabetes, metabolic syndrome and reproductive cancers^49,50^. The association between 2D:4D and age at menarche may enable detecting a high risk group and improve their outcomes. Although earlier age at menarche by itself is not an adverse health finding, it is a possible character of women who are at greater risk for reproductive challenges and morbidities. Our findings suggest digit ratio, which has been identified as a marker of early life environment, is another indicator of these women at risk. However, as mentioned by Richards, 2D:4D cannot be applied at an individual level but may be helpful in observing statistical trends over large populations^51^.

Higher 2D:4D was also associated with heavier menses bleeding and dysmenorrhea. Women with a more feminine type right 2D:4D had a higher probability to report heavier menses bleeding as compared to women with a more masculine type digit ratio of 2D:4D and women who reported having dysmenorrhea were more likely to have higher left hand 2D:4D. Heavy menstrual bleeding may lead to iron deficiency and anemia. Additionally, heavy menstrual bleeding increases the use in health care services, along with higher surgical interventions rates^52,53^. Previous studies have found an association between 2D:4D and menstrual characteristics. McIntyre *et al*. and Richards *et al*. found a significant positive association between right 2D:4D and estradiol levels across menstrual cycle^28,29^, although Klimek *et al*. found no such correlation^43^. In addition, Kaneoke *et al*. found a negative association between right 2D:4D and severity of premenstrual symptoms^27^. These findings along with our finding regarding age at menarche support the association between 2D:4D and menses characteristics. Further studies are required to support the association between 2D:4D, menses bleeding and dysmenorrhea.

A strength of our study is a direct measure of digit length, as indirect measure may distort the 2D:4D^46,54^. Another strength of the study is that since the nuchal translucency ultrasound test is recommended for all pregnant women and covered by the health maintenance organizations according to health insurance law, women from varied socioeconomic backgrounds attended the clinic and were recruited. Yet, our study is not without limitations. There is an under representation of the Bedouin population in the study sample. Previous studies have shown that this population is characterized with higher rates of lack of prenatal care and tend to begin prenatal care around mid-pregnancy^55,56^. Due to the importance of collecting data at the beginning of pregnancy, recruitment to the study was done at the end of the first trimester. Another limitation in our study is that since recruitment took place in a clinic for pregnant women, all women in our study are fertile, and only a small number of the women were fertility challenged. An under representation of extreme cases of infertility or adverse reproductive outcomes is a possibility, potentially leading to an under estimation of the true association between 2D:4D and reproductive characteristics. Additionally, external validity of the cut points for dichotomizing 2D:4D is questionable, as the ratio is population specific, and the cut points used in the current study may not be relevant in other populations. Still our finding suggest high digit ratio is associated with sub optimal reproductive characteristics, a finding that has been reported in other populations.

To conclude, there is an association between 2D:4D and sub optimal reproductive characteristics, including later age at menarche, heavier menses bleeding and dysmenorrhea. These findings support the association between 2D:4D and reproductive characteristics in women. Our findings strengthen the association between the intrauterine period and reproductive characteristics. In order to support our findings and clarify the inconsistency in the literature, further studies in other populations are required.

## Data Availability

Due to ethics committee restrictions data is not available.
